# Dementia and Parkinson’s disease diagnoses in electronic health records vs. Medicare claims data: a study of 101,980 linked patients

**DOI:** 10.1186/s12883-023-03361-w

**Published:** 2023-09-12

**Authors:** Jay B. Lusk, Sujung Choi, Amy G. Clark, Kim Johnson, Cassie B. Ford, Melissa A. Greiner, Margarethe Goetz, Brystana G. Kaufman, Richard O’Brien, Emily C. O’Brien

**Affiliations:** 1 Duke University School of Medicine, DUMC 3710, Durham, NC 27710 USA; 2 Duke University Fuqua School of Business, Durham, NC USA; 3 Department of Population Health Sciences, Duke University, Durham, NC USA; 4 Department of Neurology, Duke University, Durham, NC USA; 5 Janssen Scientific Affairs, Inc, Titusville, NJ USA; 6 Department of Psychiatry and Behavioral Sciences, Duke University, Durham, NC USA

**Keywords:** Dementia, Parkinson’s disease, Neurodegenerative disease, Electronic health records, Claims data

## Abstract

**Background:**

Medicare claims and electronic health record data are both commonly used for research and clinical practice improvement; however, it is not known how concordant diagnoses of neurodegenerative diseases (NDD, comprising dementia and Parkinson’s disease) are in these data types. Therefore, our objective was to determine the sensitivity and specificity of neurodegenerative disease (NDD) diagnoses contained in structured electronic health record (EHR) data compared to Medicare claims data.

**Methods:**

This was a retrospective cohort study of 101,980 unique patients seen at a large North Carolina health system between 2013–2017, which were linked to 100% North and South Carolina Medicare claims data, to evaluate the accuracy of diagnoses of neurodegenerative diseases in EHRs compared to Medicare claims data. Patients age > 50 who were enrolled in fee-for-service Medicare were included in the study. Patients were classified as having or not having NDD based on the presence of validated ICD-CM-9 or ICD-CM-10 codes associated with NDD or claims for prescription drugs used to treat NDD. EHR diagnoses were compared to Medicare claims diagnoses.

**Results:**

The specificity of any EHR diagnosis of NDD was 99.0%; sensitivity was 61.3%. Positive predictive value and negative predictive value were 90.8% and 94.1% respectively. Specificity of an EHR diagnosis of dementia was 99.0%, and sensitivity was 56.1%. Specificity of an EHR diagnosis of PD was 99.7%, while sensitivity was 76.1%.

**Conclusions:**

More research is needed to investigate under-documentation of NDD in electronic health records relative to Medicare claims data, which has major implications for clinical practice (particularly patient safety) and research using real-world data.

**Supplementary Information:**

The online version contains supplementary material available at 10.1186/s12883-023-03361-w.

## Introduction

Neurodegenerative diseases (NDD) are common in the United States: more than 6 million Americans age 65 and older are estimated to have Alzheimer’s disease (AD), and nearly 1 million Americans are estimated to have Parkinson’s disease (PD) [1, 2]. Alzheimer’s disease is the most common neurodegenerative disease in the United States and results in progressive, debilitating cognitive impairment leading eventually to death [3]. Parkinson’s disease is the second most common neurodegenerative disease in the United States and causes bradykinesia and tremor in addition to a host of non-motor symptoms that can also contribute to progressive disability [4]. NDD diagnoses have major implications for clinical care: patients with dementia may require alternative communication strategies, are at higher risk for social isolation, and may require significant assistance with activities of daily living; patients with PD are likewise at a greater risk of falls, a major cause of morbidity in healthcare settings [5, 6]. Furthermore, clinicians may consider alternative therapies or etiologies for patient symptoms in the context of their NDD history.

There has been an increasing interest in utilizing electronic health record (EHR) data to study NDD; however, it is not known how accurately NDD is captured within EHRs [7, 8]. EHR data can often provide more rich clinical information, as investigators can incorporate data obtained from visit notes, imaging and laboratory results, and other information that is not available in claims data. However, information recorded within one health facility’s EHR may not be available to clinicians in other facilities [9]. Additionally, it is not known how effectively information about NDD diagnoses are captured using structured EHR data elements. Center for Medicare and Medicaid Services (CMS) claims data, which rely on International Classification of Disease (ICD) codes for billing purposes, have been shown to accurately identify individuals with dementia [10]. We evaluated the concordance between EHR diagnoses of NDD and CMS claims data in a cohort of beneficiaries with available EHR data at the cross-sectional level of a large academic health system, including both inpatient and outpatient encounters across all specialties and care sites.

## Methods

### Ethical approval and consent to participate

This was a retrospective, observational study and was performed under an informed consent exemption obtained from the Duke University Institutional Review Board, protocol number 00105036. All the methods and procedures carried out in this study were in accordance with relevant guidelines and regulations. Patient privacy was protected per the stipulations in the Medicare Data Use Agreement and IRB approval, which included secure access-regulated storage of linked data, exclusion of direct identifiers from the analytic dataset, and strict adherence to cell suppression guidance.

### Data source

We retrospectively linked EHR data from the Duke University Health System (DUHS) with Medicare claims data from all North and South Carolina fee-for-service beneficiaries from 2014–2017, with a one-year lookback. Inclusion criteria for each respective study denominator year 2014–2017 were: age >  = 50 years old, living in the USA, and enrolled in fee-for-service Medicare Parts A and B and Medicare Part D on December 31 of the study denominator year and for 12 months prior and had at least one EHR encounter during the study denominator year. The pooled EHR-Medicare cohort consisted of unique beneficiaries in any 2014–2017 study denominator year based on the earliest year identified. These inclusion criteria were selected to ensure that patients had sufficient enrollment in Medicare to ascertain baseline characteristics and comorbidities.

### Data linkage

Data were linked by linking patient IDs from Duke EHR data to beneficiary IDs from CMS data. Linkage was accomplished by requesting a crosswalk file from CMS which identified CMS beneficiary IDs corresponding to all patients with records available in Duke EHR data. Date of birth and sex were then used to validate identifier matching. EHR data from DUHS were directly obtained from the EPIC data server and then standardized to the PCORNet Common Data Model, a data specification that defines a standard organization and representation of EHR data for use in distributed, network-based research. For our study, we used EHR data from the DIAGNOSIS, PRESCRIBING, and DISPENSING tables. Multiple diagnoses and/or prescriptions for a given encounter are represented as additional records within the appropriate table.

### NDD identification algorithm in claims data

We used a previously validated algorithm to identify beneficiaries with evidence of NDD based on 1) an ICD-CM-9 or ICD-CM-10 diagnosis code in any position on an inpatient, outpatient, carrier, skilled nursing facility or home health claim, or 2) a claim for a prescription drug to treat dementia or PD between 1/1/2013 and 12/31/2017 (Additional file 1) [11]. The earliest diagnosis code or drug prescription found was used as the index NDD diagnosis for both data sources.

### NDD identification algorithm in EHR data

Patients were identified to have NDD if 1) any ICD-CM-9 or ICD-CM-10 code (Additional file 1) of NDD was found in the encounter diagnosis table or billing diagnosis list in the EHR, or 2) at least one prescription record for a drug used to treat dementia or PD was found in the prescribing or dispensing tables between 1/1/2013 and 12/31/2017. EHR data is available for any inpatient, outpatient, or home health visit within the Duke University Health System, but is not available for visits at other healthcare organizations.

### Statistical analysis

Baseline characteristics were described using medians and interquartile ranges for continuous variables and counts with percentages for categorical variables. Medicare-based algorithms were defined as the reference standard and sensitivity, specificity, negative predictive value, and positive predictive value of EHR derived NDD were calculated. We defined Medicare diagnoses as the reference standard in our study given prior literature demonstrating that claims-based algorithms were highly accurate at identifying patients with NDD compared to a gold standard of clinically adjudicated dementia [10, 12]. A secondary analysis restricted to beneficiaries with at least two outpatient encounters was performed to exclude patients who were only seen on one occasion. Analysis was conducted in SAS 9.4. This was an exploratory analysis, and accordingly no *p*-values were reported for comparisons shown.

## Results

**Fig. 1:**
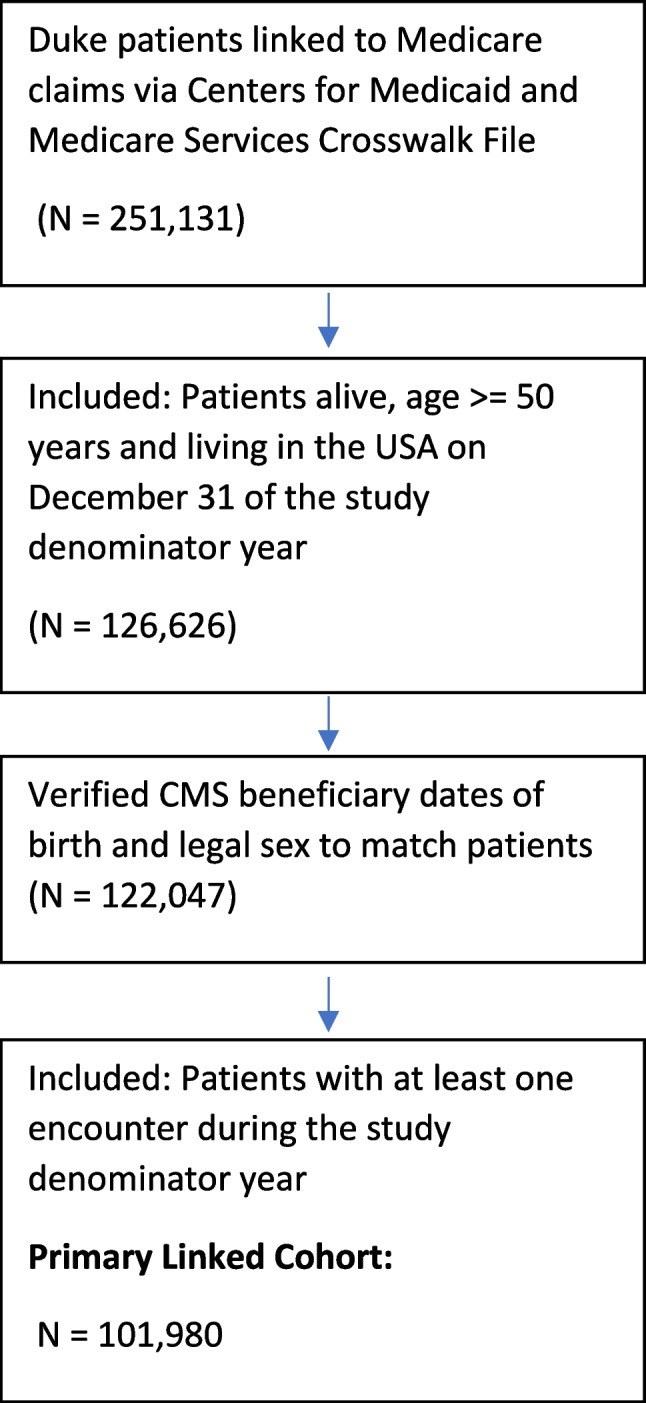
Flow diagram showing the data linkage and cohort creation process

One hundred one thousand, nine hundred eighty unique patients were included in the primary linked EHR-Medicare cohort (Table 1). The data linkage process is visualized in Fig. 1. Median age was 70 (IQR: 66, 76). The cohort was 57.4% female, 75.3% White, 20.2% Black; 19.9% of patients were dually-eligible for Medicaid and Medicare, and 27.4% resided in a rural area. Prevalent rates of NDD diagnosis in each study year and in the overall population of patients ever diagnosed were higher in Medicare claims than EHR data (Table 2). Similar patterns were observed for dementia, where 8,156 (8.0%) pooled patients were identified as having ever having a dementia diagnosis in Medicare data, compared with only 4,858 (4.8%) patients in the EHR. Finally, 1,933 (1.9%) pooled patients had a Medicare diagnosis of PD, compared with 1,678 (1.7%) patients with an EHR diagnosis of PD (Table 2).

**Table 1:** Baseline characteristics of the linked electronic health records-Medicare cohort

	**2014**	**2015**	**2016**	**2017**	**Pooled (2014–2017)**^a^
N	50,083	57,610	61,301	63,492	101,980
Demographics					
Age, years, Median (Quartile 1, Quartile 3)	71.0 (67.0, 77.0)	72.0 (67.0, 77.0)	72.0 (68.0, 78.0)	72.0 (68.0, 78.0)	70.0 (66.0, 76.0)
Age categories					
50–64	19,847 (39.6%)	22,091 (38.3%)	22,669 (37.0%)	22,399 (35.3%)	46,392 (45.5%)
65–74	12,668 (25.3%)	14,698 (25.5%)	16,170 (26.4%)	17,375 (27.4%)	24,022 (23.6%)
75–84	8,644 (17.3%)	10,079 (17.5%)	10,909 (17.8%)	11,775 (18.5%)	15,781 (15.5%)
85 +	8,924 (17.8%)	10,742 (18.6%)	11,553 (18.8%)	11,943 (18.8%)	15,785 (15.5%)
Sex, Female	29,188 (58.3%)	33,482 (58.1%)	35,527 (58.0%)	36,657 (57.7%)	58,517 (57.4%)
Race/Ethnicity					
White	37,615 (75.1%)	43,751 (75.9%)	46,518 (75.9%)	48,406 (76.2%)	76,772 (75.3%)
Black	10,504 (21.0%)	11,471 (19.9%)	12,037 (19.6%)	12,081 (19.0%)	20,574 (20.2%)
Other	1,964 (3.9%)	2,388 (4.1%)	2,746 (4.5%)	3,005 (4.7%)	4,634 (4.5%)
Dual Medicaid Eligibility	10,050 (20.1%)	10,754 (18.7%)	10,938 (17.8%)	10,854 (17.1%)	20,336 (19.9%)
Rural location	13,898 (27.7%)	14,712 (25.5%)	15,196 (24.8%)	15,440 (24.3%)	27,935 (27.4%)
State					
North Carolina	48,785 (97.4%)	56,140 (97.4%)	59,773 (97.5%)	61,871 (97.4%)	98,683 (96.8%)
South Carolina	1,298 (2.6%)	1,470 (2.6%)	1,528 (2.5%)	1,621 (2.6%)	3,297 (3.2%)

^a^Unique beneficiaries in any 2014–2017 denominator defined based on earliest denominator year

**Table 2:** Prevalent percentage rates of neurodegenerative disease in the linked Electronic Health Records (EHR)-Medicare cohort in 2014–2017 per 100 person-years

**Disease Group**	**2014**		**2015**		**2016**		**2017**		**Pooled (2014–2017)**a	
	**Medicare**	**EHR**	**Medicare**	**EHR**	**Medicare**	**EHR**	**Medicare**	**EHR**	**Medicare Ever Diagnosed**	**EHR Ever Diagnosed**
**Neurodegenerative Disease (N, %)**	4,506 (9.0)	3,178 (6.4)	5,808 (10.1)	4,052 (7.0)	7,111 (11.6)	4,986 (8.1)	7,936 (12.5)	5,675 (8.9)	10,031 (9.8)	6,472 (6.4)
**Dementia**	3,591 (7.2)	2,362 (4.7)	4,655 (8.1)	3,018 (5.2)	5,823 (9.5)	3,814 (6.2)	6,578 (10.4)	4,444 (7.0)	8,156 (8.0)	4,858 (4.8)
**Parkinson's Disease**	947 (1.9)	846 (1.7)	1,183 (2.1)	1,061 (1.8)	1,317 (2.2)	1,206 (2.0)	1,383 (2.2)	1,260 (2.0)	1,933 (1.9)	1,678 (1.7)

^a^Unique pooled beneficiaries from all study denominator years (2014–2017)

Compared with Medicare claims, EHR data were highly specific for identifying NDD (99.0%), but only moderately sensitive (61.3%; Table 3). Positive predictive value (PPV) was 90.8% and negative predictive value (NPV) was 94.1%. EHR data were also highly specific for dementia, (99.0%), while sensitivity for dementia was 56.1%. Finally, the specificity of an EHR diagnosis of PD was 99.7%, while sensitivity was 76.1%. In a secondary analysis including only patients with at least two encounters, specificity estimates were similar, but sensitivity estimates increased slightly for NDD (67.1%), dementia (61.7%), and PD (81.4%) (Table 3).

**Table 3:** Sensitivity, specificity, positive predictive value, and negative predictive value for EHR diagnoses compared to Medicare reference standard diagnoses for patients seen at least once and at least twice

**Diagnosis (One-Visit)**	**Sensitivity (One-Visit)**	**Specificity (One-Visit)**	**Positive Predictive Value (One-Visit)**	**Negative Predictive Value (One-Visit)**
*Neurodegenerative Disease*	61.3%	99.0%	90.8%	94.1%
*Dementia*	56.1%	99.0%	88.0%	94.6%
*Parkinson Disease*	76.1%	99.7%	86.9%	99.4%
**Diagnosis (Two-Visit)**	**Sensitivity (Two-Visit)**	**Specificity (Two-Visit)**	**Positive Predictive Value (Two-Visit)**	**Negative Predictive Value (Two-Visit)**
*Neurodegenerative Disease*	67.1%	98.7%	88.6%	95.2%
*Dementia*	61.1%	98.5%	81.3%	95.9%
*Parkinson Disease*	81.5%	99.7%	86.1%	99.6%

## Discussion

EHR data hold promise for research on real-world NDD populations, but information on validity of EHR-based diagnoses is limited. We investigated the concordance between EHR diagnosis of neurodegenerative diseases and CMS claims diagnosis of neurodegenerative diseases in a large population of Medicare beneficiaries seen at an academic health system over a four-year period. Our main findings were: 1) EHRs were only moderately sensitive for detecting NDD compared to Medicare claims data, 2) EHRs were comparatively more sensitive in documenting PD compared to dementia (roughly 75% for PD vs. 56% for dementia); 3) EHR diagnoses of any NDD, dementia, and PD were all highly specific for CMS diagnosis of equivalent pathology.

There are several potential reasons why NDD may not be detectable in EHR data. It is possible that some patients received care at the health system only once, and their clinical history of NDD was not documented at that single encounter. Indeed, we observed a modest increase in sensitivity of dementia diagnosis when restricting the population to those with 2 outpatient healthcare encounters in the prior year. Alternatively, it is possible that patients or family members, especially in cases of mild NDD, may not have volunteered information about NDD diagnoses, and clinicians may not have asked directly about cognitive status [13, 14]. Notably, the EHR was more sensitive for identifying PD than dementia, perhaps because PD is a more directly visible diagnosis or because PD medications are more frequently used than dementia medications or more closely monitored by clinicians.

There are several implications for the relatively low sensitivity of EHR data for NDD diagnoses. First, health systems should be aware of safety implications arising from clinical staff not being aware of NDD diagnoses: patients with cognitive impairment may be at higher risk of delirium and falls, for example, two common healthcare-associated adverse events. Additionally, researchers should be aware that utilizing EHR data alone, even from a large, integrated health system, without access to claims from all providers outside the system, may fail to identify many patients with NDD and could potentially introduce bias into such studies.

The high specificity of EHR diagnoses of NDD, dementia, or PD suggest that, for many clinical and research purposes, positive EHR NDD diagnoses can be assumed to be accurate compared with CMS data. Strengths of the study include its large size, fully real-world data utilization, and inclusion of a diversity of sites, clinics, and specialties. Notably, our study did not stratify by specialty or visit type, but there is likely substantial specialty-specific variation in diagnosis coding: for example, it is reasonable to expect that neurologists might diagnose Parkinson’s disease at greater rates than other specialists. Future work to investigate variation in the sensitivity and specificity of these diagnosis codes by specialty and visit type would be warranted. However, while there is generally good justification from prior literature for using CMS data as a reference standard, some misclassification of patients, especially those with mild cognitive impairment, is possible [12]. Claims data reflect the realities of billing practices rather than underlying clinical care provided, which may further limit the utility of using claims data as the reference standard; however, prior literature suggests that claims data is primarily limited by a lack of sensitivity compared to gold standard clinical diagnosis, rather than lack of specificity [10, 12]. Since our study found that EHR data were poorly sensitive compared to Medicare data, this increases the likelihood that EHR data is also poorly sensitive compared to gold standard clinical diagnosis. Furthermore, this study utilized structured EHR data, so findings may not be generalizable to unstructured data sources or outside the health system studied. Lack of use of unstructured data fields may limit the sensitivity of EHR data in identifying NDD; however, claims data also contains no unstructured data sources, and therefore we would not expect a systematic bias in our results due to lack of use of unstructured data sources. Generalizability is a major limitation of our study: it is possible that diagnosis and practice patterns at other health systems in other geographies or with differing operational structure would meaningfully affect the rate at which NDD is documented in EHR data. Finally, our study did not explore variation in EDD diagnosis coding patterns between different physicians, and it is possible that some physicians may have more accurate coding patterns than others: future work could investigate this question.

## Conclusions

Structured electronic health record data were highly specific but only moderately sensitive for identifying dementia and Parkinson’s disease compared to Medicare claims data. Further work to improve EHR documentation of NDD to support clinical care and population-based research is needed.

### Supplementary Information

**Additional file 1. **Neurodegenerative disease diagnosis codes and prescription drugs used to identify neurodegenerative diseases.

## Data Availability

Data may be requested from the Centers for Medicare and Medicaid Services by contacting the Research Data Assistance Center (ResDAC).

## Abbreviations

NDD: Neurodegenerative Diseases
EHR: Electronic Health Records
PD: Parkinson’s Disease
