# Supplementary material for: Dementia and Parkinson’s disease diagnoses in electronic health records vs. Medicare claims data: a study of 101,980 linked patients
Source: BMC Neurol. 2023 Sep 12;23:325. doi: 10.1186/s12883-023-03361-w (PMC10496225; doi:10.1186/s12883-023-03361-w)
Supplement: Supplementary file 1 — Additional file 1. Neurodegenerative disease diagnosis codes and prescription drugs used to identify neurodegenerative diseases. [file 12883_2023_3361_MOESM1_ESM.docx]

**Additional file 1. Neurodegenerative disease diagnosis codes and prescription drugs used to identify neurodegenerative diseases**

|  | *ICD-9-CM* Codes | *ICD-10-CM* Codes |
| --- | --- | --- |
|  | HEDIS NCQA + Goodman codes:  046.11, 046.19, 290.0, 290.10, 290.11, 290.12, 290.13, 290.20, 290.21, 290.3, 290.40, 290.41, 290.42, 290.43, 290.8, 290.9, 291.2, 292.82, 294.1, 294.10, 294.11, 294.20, 294.21, 294.8, 331.0, 331.1, 331.2, 331.82 (dementia with parkinsonism), 333.4, 797  Codes only from Goodman, et al:  046.11  Creutzfeldt-Jakob disease, variant  046.19   Creutzfeldt-Jakob disease, other and unspecified  291.2     Alcohol induced persisting dementia  292.82   Drug induced persisting dementia  294.1   Dementia in conditions classified elsewhere (Not a valid code)  331.11   Pick’s disease  331.19  Other frontotemporal dementia  333.4     Huntington's chorea  797        Senility w/o psychosis | HEDIS NCQA + Goodman codes:  G30.0x, G30.1x, G30.8x, G30.9x, F03.9x, G31.0x, G31.1x, G31.2x, G31.8x (G31.83: dementia with parkinsonism), G31.9x, F01.50, F01.51, F02.80, F02.81, F03.91, F05, F06.8, F10.27, A81.00, A81.01, A81.09, , F18.97, F19.97, G10, R41.81, F13.27,  F13.97, F18.17,  F18.27,  F19.17,  F19.27 |
| Parkinson’s Disease | 332 (Parkinson’s disease), 332.0 (Paralysis agitans) | G20 (Parkinson’s disease) |

| **Generic Medication Names** | **Definition/Assessment** |
| --- | --- |
| **Dementia drugs** |  |
| Donepezil | Part D claim for generic drug name like ‘%DONEPEZIL%’ within 365 days prior to and 90 days following index date. |
| Memantine | Part D claim for generic drug name like ‘%MEMANTINE%’ within 365 days prior to and 90 days following index date. |
| Rivastigmine | Part D claim for generic drug name like ‘%RIVASTIGMINE%’ within 365 days prior to and 90 days following index date. |
| Galantamine | Part D claim for generic drug name like ‘%GALANTAMINE%’ within 365 days prior to and 90 days following index date. |
| **Parkinson’s drugs** |  |
| Levodopa | Part D claim for generic drug name like ‘%LEVODOPA%’ within 365 days prior to and 90 days following index date. |
| Carbidopa | Part D claim for generic drug name like ‘%CARBIDOPA%’ within 365 days prior to and 90 days following index date. |
| Entacapone | Part D claim for generic drug name like ‘%ENTACAPONE%’ within 365 days prior to and 90 days following index date. |
|  |  |
